# Retraction to Constructing interfacial potential barrier via a gradient configuration: an effective method to enhance energy-filtering effect

**DOI:** 10.1093/nsr/nwaa256

**Published:** 2020-12-18

**Authors:** Zizhen Lin, Yinshi Li, Ya-Ling He

‘Constructing interfacial potential barrier via a gradient configuration: an effective method to enhance energy-filtering effect’ (*National Science Review*, published online: 3 September 2020, doi: 10.1093/nsr/nwaa223). The authors have alerted us that recent experimental work conducted indicates that some expiations of this manuscript need to be further discussed. In agreement with the authors, editors and publisher, *National Science Review* has therefore decided to retract the paper. The journal would like to apologise for any inconvenience caused to the reader.

